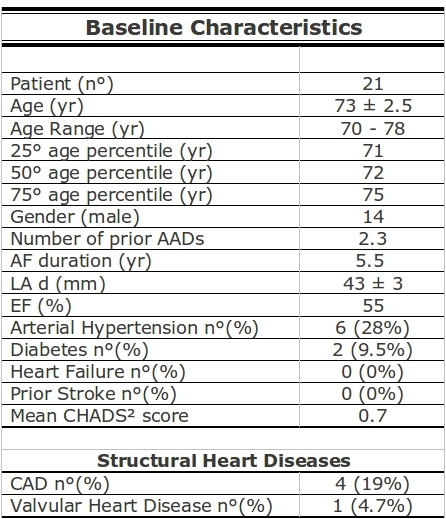# Cryoballoon Ablation for Paroxysmal Atrial Fibrillation in Septuagenarians: a Prospective Study

**Published:** 2010-09-05

**Authors:** Gian Battista Chierchia, Lucio Capulzini, Carlo de Asmundis, Andrea Sarkozy, Antonio Sorgente, Yoshinao Yazaki, Stephan-Andreas Muller-Burri, Gaetano Paparella, Marc Lameir, Fatih Bayrak, Roberto De Ponti, Pedro Brugada

**Affiliations:** 1 Heart Rhythm Management Centre, UZ Brussel-VUB, Brussels, Belgium; 2 Department of Heart Sciences, Ospedale di Circolo and Fondazione Macchi, Varese, University of Insubria, Varese, Italy

**Keywords:** cryoballoon, elderly, atrial fibrillation

## Abstract

**Aims:**

To evaluate the effects of pulmonary vein isolation (PVI) in terms of feasibility, safety and success rate on a midterm follow-up period in septuagenarians undergoing ablation with the Arctic Front Cryoballoon for atrial fibrillation (AF).

**Methods and Results:**

We prospectively enrolled 21 patients aged 70 years or older (14 male; age 73 ± 2.5 years) elected to circumferential PVI with the 28mm cryoballoon for symptomatic drug resistant paroxysmal AF. A total number of 82 pulmonary veins (PV) were evidenced. Successful isolation could be obtained in all 82 (100%) PV ostia at the end of procedure. No major complication occurred during procedure. At a mean follow-up of 11.5 ± 4.7 months following ablation, 62% of patients did not present recurrence of atrial arrhythmias.

**Conclusion:**

Cryoballoon ablation may be feasible and safe in older patients. Moreover a large proportion of the latter did not present AF recurrence during follow-up.

## Introduction

Transcatheter ablation has become a common and efficient technique in the treatment of drug resistant atrial fibrillation (AF). Since ectopic beats originating from the pulmonary veins (PV) have been shown to be the main trigger initiating AF [1,2], electrical isolation of these venous structures has certainly become the most widely accepted endpoint when performing this procedure. Traditionally, PV isolation (PVI) is performed by creating radiofrequency (RF) energy lesions with of a focal tip catheter. Since AF is the most common arrhythmia encountered in clinical practice and its incidence is known to augment with age, reaching more than 7% in septuagenarians [3,4], older patients might represent an interesting target group for PVI procedures. However, as patients get older, fibrosis and fat deposits infiltrate the myocardium leading to an alteration in electroanatomical atrial substrate. Conceptually this might render older patients less responsive to AF ablation. Furthermore, elderly patients present more frequently co-morbidities such as hypertension or congestive heart failure. Also, an age of over 75 years is known to be an independent risk factor for embolic events. This might lead electrophysiologists to be more cautious when proceeding to ablations in the left atrium with a large amount of lesions in these individuals. Fortunately, recently published data seems to indicate that AF ablation using RF is safe and feasible in elderly patients [5,6]. Moreover, this procedure might also be very effective in this patient population reaching success rates of more than 80% at one year follow-up. Novel ablation catheters designed as balloons have proven very effective in achieving PVI. Specifically, the cryoballoon (Arctic Front, Medtronic) using cryothermic ablation energy has been reported to acutely achieve PVI in more than 90% of veins [7,8]. In the present study, we prospectively evaluated the effects of PVI using the cryoballoon in terms of feasibility and safety in a series of elderly patients (aged ≥70 years old). To our knowledge, this is the first study conducted on such an old poulation undergoing cryoballoon ablation. In fact, all previous reports available in the literature included much younger series of patients [7-9].

## Methods

### Patient characteristics

In a larger series of consecutive patients undergoing PVI with the cryoballoon, all patients aged ≥70 years old were prospectively enrolled in our study. A total of 21 patients (14 male; mean age 73 ± 2,5 years) were included (see Table 1 for baseline characteristics). Four patients underwent a previous RF PVI procedure using a point by point with electroanatomical mapping guidance in another center, with early AF recurrence following the ablation. Reports from the above mentioned 4 procedures indicate that PVI was successfully achieved in all veins by the use of a circular mapping catheter in the PV ostia during ablation All individuals signed an informed consent prior to procedure according to our institution's guidelines. The study was approved by the institutional review board. Inclusion criteria were based on the frequent occurrence of recurrent episodes of AF, in spite of treatment with at least 2 antiarrhythmic drugs and patient age ≥70 years. To exclude the presence of thrombi in the left atrial appendage, all patients underwent 2D transesophageal echocardiography (TEE) the day before the procedure, along with a transthoracic echocardiography (TTE) enabling assessment of left atrial (LA) dimensions, left ventricular and valvular function. Exclusion criteria were the presence of a LA thrombus, uncontrolled severe heart failure, or contraindications to general anesthesia.

### Ablation procedure

**Figure 1:**
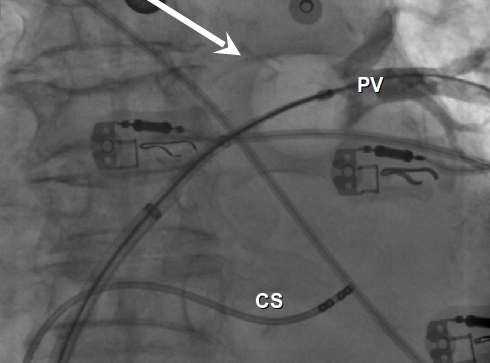
Fluoroscopy angiography of the left superior pulmonary vein during occlusion with the cryoballoon(arrow). Quadripolar catheter in the coronary sinus. PV:pulmonary vein; CS: coronary sinus.

The cryoballoon ablation procedure was carried out as previously described in detail [7]. Ablation was performed under general anesthesia. Briefly, after gaining left atrial access, selective angiograms of the PVs were performed and baseline electrical information using a circular mapping catheter (Lasso, Biosense Webster, Inc., Diamond Bar CA, USA) was aquired. Then a 28 mm double walled cryoballoon (Arctic Front, Medtronic, Minneapolis) was inflated in the LA and positioned sequentially in the PV ostium of each vein. Occlusion was considered to have been achieved when selective contrast injection showed total contrast retention with no backflow to the atrium (Figure 1). A minimum of 2 cryoenergy applications were performed per vein. In order to avoid phrenic nerve palsy during cryoablation in the RSPV, a quadripolar catheter was placed in the superior vena cava and pacing was achieved by pacing the ipsilateral phrenic nerve and ensuring diaphragmatic contraction. During the whole procedure activated clotting time was maintained over 300 seconds by supplementing heparin infusion, as required.

### Assessment of PV isolation

We recorded PV activity before and after ablation. Effective PV isolation was considered to have been obtained when all PV potentials were abolished or dissociated from atrial activity, and bidirectional block was documented. Entry block was demonstrated by the absence of PV activity while pacing in the distal CS. Exit block was verified by pacing from each bipolar pair of the mapping catheter in the PV at 10 mA and 2 ms pulse width. Thirty minutes following electrical isolation, the circular mapping catheter was placed again sequentially in each PV ostium to verify persistence of the result.

### Follow-up

All patients underwent physical examination and 24 hour Holter recordings at 1, 3, 6 days and a 5 day loop Holter recording 12 months after the procedure. Additional Holter monitoring was performed if arrhythmic symptoms occurred. We considered a post-ablation blanking period of 2 months. Antiarrythmic drugs were continued during the blanking period and then stopped.

### Statistical analysis

Continuous variables are expressed as mean ± SD. Categorical variables are expressed as percentages.

## Results

Mean total procedure time was 129 ±  22 minutes (from skin puncture to complete sheath extraction from the groin). Mean total fluoroscopy time was 26 ±  6 min. Mean left atrial size was 43 ±  3 mm. Mean duration of AF before procedure was 5.5 years. Structural heart disease was present in 5 patients (coronary artery disease=4; Valvular heart disease=1). Arterial hypertension under medical treatment was noted in 6 patients. Two patients presented with history of diabetes mellitus. Four patients exhibited a CHADS2 score of ≥2. During all procedures patients were in sinus rhythm and none developed atrial fibrillation at any stage. A total number of 82 PVs were targeted. Two discrete left sided common ostia were found. Thanks to the steerability of both the FlexCath (Cryocath, Montreal, Quebec, Canada) and the Lasso (Biosense Webster, Inc., Diamond Bar CA, USA), it was possible to map all PV ostia. Potentials were present in all PV ostia before ablation in patients that did not have a previous PVI. In the 4 patients that underwent a previous PVI procedure PV potentials were present in ≥3 veins (2 patients exhibited reconnection in 3 veins and the other 2 in all 4 veins). All veins were targeted with the cryoballoon. Cryoballoon applications were also performed in the 2 veins exhibiting no baseline electrical activity because of the potential beneficial overlapping effect [9] on the ipsilateral interpulmonary carina. Successful occlusion could be obtained in 76 (92.7%) PVs. In 12 PVs we performed more than 2 CBA applications as optimal occlusion could not be reached with the first two. After a mean of 2.3 (range 2-4) cryoballoon applications, PV isolation with bidirectional block could be demonstrated in 78 (95%) of veins after cryoballoon applications  at first testing after ablation. Additional lesions with a cryothermal focal ablation catheter (Freezor Max, Medtronic, Minnesota, USA)  were needed to isolate the remaining 5% of veins (1 left superior PV (two applications on the LAA-LSPV ridge), 1 left inferior PV (single application in the posteroinferior region) and 2 right inferior PV (single application in the antero-inferior region; three applications in the inferior region)) in three different patients. After 30 minutes observational time, recovery occurred only in 2 (2.4%) right inferior PV. A single focal application in each vein led to their isolation.  At the end of the procedure all 82 (100 %) veins were isolated.

### Adverse events

Transient phrenic nerve palsy was observed in 3 patients when delivering energy in the RSPV (respectively after 156, 217 and 239 sec after starting the first cryoapplication). Diaphragmatic contraction completely recovered before the end of the procedure. Two patients experienced groin hematomas in the site of puncture. No patient experienced cerebrovascular injury, nor pericardial effusion causing acute hemodynamic impairement.

### Follow-up

Clinical examination, baseline ECG and 24 hour ECG Holter registrations were performed at 1, 3 and 6 months after the procedure. A 5 day loop holter was systematically performed in all patients at 12 months after ablation. At a mean follow-up of 11,5 ± 4,7 months, 8 patients (38%) experienced arrhythmic symptoms. During the first two months, 2 patients experienced short episodes of sustained AF (documented on Holter monitoring). During the rest of the follow-up none of these patients had AF recurrence. Typical right atrial flutter was documented as the clinical arrhythmia in one patient still experiencing symptoms. Following cavotricuspid isthmus ablation, the patient was free of arrythmias. Five patients experienced persistence of AF episodes during follow-up. Persistence of sinus rhythm could be achieved with single antiarrythmic drug (AAD) treatment in 4 patients. One patient was still symptomatic despite medical treatment with AADs. Due to failure of both drug therapy and ablation in this patient, a repeat procedure was proposed but was refused.

## Discussion

AF is the most frequently encountered arrhythmia in occidental society reaching epidemic proportions in the elderly population. It is in fact estimated that its incidence reaches more than 7% in individuals between 70 and 80 years old [3,4]. Drug treatment of AF is known to be often ineffective in maintaining sinus rhythm. Consequently, catheter ablation is increasingly being performed in electrophysiology laboratories around the world as it has proven an efficient tool to treat this arrhythmia [10]. As age related senescence is known to alter the pharmacokinetics of medical treatment making the metabolism of anti-arrhythmic agents less predictable (thus increasing the probability of side effects), [11,12] ablation might play a key role in preventing arrhythmic recurrence also in older patients. Furthermore, as the elderly represent the fastest growing segment of our population, studies focused on PVI in these patients have recently been reported in the literature. Corrado et al [5], described a surprisingly high rate of ablation success in a large cohort of patients over 70 years old following 2 ablation procedures. In fact 82% of patients were able to maintain sinus rhythm off AAD's with an additional 12% if medical therapy was added. Zado et al [6] report similar findings in their single center observation. A retrospective analysis of over 7 years concluded that it was possible to reach AF control in a high percentage of previously symptomatic despite drug treatment elderly patients (84% for patients aged between 65 and 74 years; 86% for patients over 75 years old). More importantly ablation in the elderly was not associated to increased risk of complications in comparison to younger individuals. However, older patients were more likely to remain on anti-arrhythmic medication to achieve long-term control of the arrhythmia. The 2 abovementioned studies, however bare the obvious limitation of both being retrospective analyses. A recent prospective observation by Traub et al [13] reported a success rate of 60% at one year after one procedure in patients over 70 years of age. Similarly to Zado et al the authors concluded that septuagenarians were more likely to continue anti-arrhythmic medication following ablation and that PVI was safe and feasible in this patient population. These studies were all conducted on patients undergoing PVI with RF energy. Novel ablation catheters designed as balloons have proven very effective in achieving PVI. Specifically, the cryoballoon (Arctic Front, Medtronic) using cryothermic ablation energy has been reported to acutely achieve PVI in more than 90% of veins [7-9]. To our knowledge the present study is the first prospective observation in terms of safety, feasibility and midterm outcome in a series of septuagenarians undergoing AF ablation with the cryoballoon. The study's main findings are that this technique might be safe and feasible in elderly patients achieving a high rate of electrical isolation using the cryoballoon approach. In terms of feasibility we observed electrical isolation in all veins at the end of the procedure. In fact after a mean 2.3 balloon applications per vein electrical isolation could be documented in 95% of venous ostia. Additional focal applications achieved electrical isolation in the remaining 5%. Also, cryoballoon PVI might be safe in elderly patients. The only complication that required additional hospitalization days was a right sided groin hematoma in one patient. The patient was not dismissed immediately for purely observational reasons. This adverse event might be considered more procedure than device related. As described in other studies, phrenic nerve palsy during cryoballoon is mostly reversible. In our series the large 28 mm balloon was used systematically. This probably resulted in more proximal lesions in the RSPV antrum with a lower possibility of phrenic nerve injury. Furthermore no CVA was observed. The cryothermal lesion itself might be part of the reason, as it is known to be less thrombogenic than RF [14]. However, this is a purely speculative statement and is strongly limited by the size of our population. Thus, larger trials evaluating CVA rate following cryoballoon ablation in the elderly are warranted. At a mean follow-up of 11.5 months, 62% of patients were symptom free after ablation only. This proportion is in the range of success of a single AF ablation procedure in younger patients [7,8]. This might indicate that even in the setting of a more remodelled atrial myocardium, triggers in the PVs might still be the main cause of paroxysmal AF in the elderly.  In our report 6 patients (29%) required further treatment following ablation. This finding is similar to the one reported by Zado et al stating that elderly patients are more liable to remain on antiarrythmic drugs to achieve AF control following RF ablation. However, conversely to younger patients in which AF freedom without drug therapy might be the primary clinical endpoint, in an elderly population affected by drug resistant AF, symptom relief following ablation might be considered a positive outcome even if drug therapy is not discontinued.

## Limitations

Our study bares the limit of a single center study with a relatively small population. Furthermore, in our series the mean left trial diameter was roughly 43 mm and structural heart disease was present in only 5 patients (24%) reflecting a relatively "healthy" population despite the age. Larger atria and higher comorbidity might have led to a less favourable outcome. Moreover, no patient in our series exhibited abnormal anatomical features, such as extremely large common ostia or adjunctive veins with uncommon ostial position, which might have rendered PV isolation very difficult with the cryoballoon.

## Conclusion

Cryoballoon ablation may be feasible and safe in older patients. Moreover a large proportion did not present AF recurrence during follow-up. Studies with larger number of elderly patients are warranted in order to confirm our findings.

## Figures and Tables

**Table 1:**